# Booster effect of the third dose of SARS-CoV-2 mRNA vaccine in Japanese kidney transplant recipients

**DOI:** 10.1038/s41598-023-36998-1

**Published:** 2023-06-20

**Authors:** Mayuko Kawabe, Takafumi Kuroda, Izumi Yamamoto, Akimitsu Kobayashi, Yutaro Ohki, Ayaka Hayashi, Fumihiko Urabe, Jun Miki, Hiroki Yamada, Takahiro Kimura, Nanae Matsuo, Yudo Tanno, Tetsuya Horino, Ichiro Ohkido, Hiroyasu Yamamoto, Takashi Yokoo

**Affiliations:** 1 Division of Nephrology and Hypertension, Department of Internal Medicine, The Jikei University School of Medicine, Tokyo, Japan; 2 Department of Urology, The Jikei University School of Medicine, Tokyo, Japan; 3 Department of Infectious Disease and Infection Control, The Jikei University School of Medicine, Tokyo, Japan

**Keywords:** Immunology, Microbiology, Nephrology, Urology

## Abstract

The humoral response of kidney transplant recipients (KTR) to the mRNA vaccine against severe acute respiratory syndrome coronavirus 2 (SARS-CoV-2) is generally poor. We evaluated the booster effect of the third dose (D3) of two SARS-CoV-2 mRNA vaccines 6 months after the second dose (D2) in Japanese KTR. The anti-spike (anti-S) antibody titer 1 and 3 months after the D3 was evaluated in 82 Japanese KTR. The primary endpoint was the seropositivity rate, and factors associated with the lack of a response were evaluated in a logistic regression model. Overall, the anti-S antibody seropositivity rate 1 and 3 months after the D3 was 74.7% and 76.0%. The anti-S antibody titers after the first and second doses were higher in patients vaccinated with the mRNA-1273 than with the BNT162b2 vaccine. Among the 38 KTR who were seronegative 5 months after the D2, 18 (47.4%) became seropositive following the D3. Factors associated with a non-response were mycophenolic acid dose, post-transplant duration, hemoglobin, and lymphocyte count. A humoral response 1 and 3 months after the D3 was obtained in ~ 75% of KTR, but 20% were non-responders. Additional studies are needed to clarify the factors hindering a vaccine response.

## Introduction

COVID-19 is an infection caused by severe acute respiratory syndrome coronavirus 2 (SARS-CoV-2)^1^. The mortality rate for solid-organ transplant recipients in Japan is 2.61% (30/1148 patients: heart 13, lung 21, liver 237, kidney 875, and pancreas 1)^2^, which is lower than the 22–32% reported in other countries^3,4^. However, compared to the overall COVID-19-related mortality rate in Japan of 0.3%^5^, this rate is very high. Consequently, vaccination against COVID-19 in Japanese kidney transplant recipients (KTR) is an important step to prevent infection, severe disease, and mortality in this population. The first SARS-CoV-2 variants of concern were the alpha and gamma strains but they were eventually replaced by omicron strains, which are currently responsible for nearly all infections in Japan (January 2022: BA.1, May 2021: BA.2, July 2022: BA.5)^6^. Additional vaccine doses have been shown to induce a humoral response against omicron strains but the response is lower than to the wild-type^7,8^. In Japan, the BNT162b2 and mRNA-1273 vaccines were approved on February 14, 2021, and May 21, 2021, respectively. The current policy is to administer the same vaccine for the first and second doses (D1 and D2) and then a third dose (D3) 6 months after the D2. Previously, we reported that the anti-S SARS-CoV-2 IgG antibody titer 5 months (149.2 ± 45.5 days) after the D2 was 35.3 AU/mL (interquartile range [IQR] 3.8–159.7), but an appropriate humoral response was determined in only 47.8% of vaccinated KTR^9^. In the present study, we evaluated the booster effect of the D3 in Japanese KTR. Hence, this is the second report, related to our previous report^9^.

## Materials and methods

### Patients

The study period for this prospective observational study was from January 2021 to December 2022. The study subjects were 94 KTR who received two doses of SARS-CoV-2 mRNA vaccine as previously reported^9^ and 3 KTR who subsequently participated in this study, for a total of 97 KTR. Patient enrollment criteria were kidney transplant recipients aged 20 years or older attending Jikei University Hospital as outpatients. Patient exclusion criteria were as follows.

**Figure 1:**
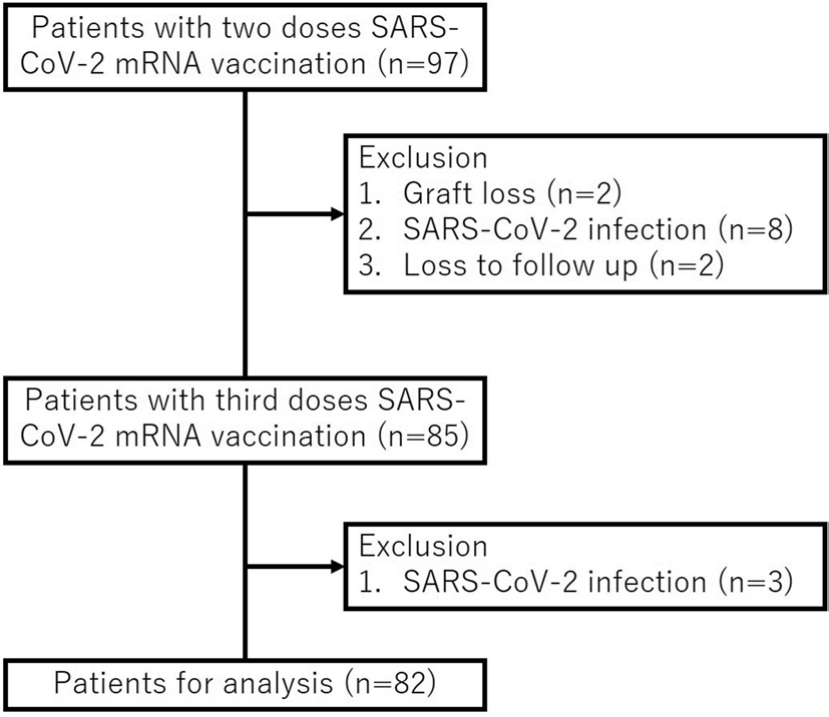
Study population.

Two patients with graft loss during the observation period, eight patients with COVID-19, and two patients who were lost to follow-up were excluded. The remaining 85 patients received the D3 of the SARS-CoV-2 mRNA vaccine, but 3 patients who developed COVID-19 within 3 months of vaccination were excluded (One of the 3 was negative for antibody titer at 5 months after D2, the other 2 were positive). Thus, data from 82 patients were analyzed (Fig. 1). In 70 patients, the D1 and D2 consisted of BNT162b2 and in 12 patients mRNA-1273. The D3 consisted of BNT162b2 in 43 patients and mRNA-1273 in 39 patients. Blood samples were collected at the time of the hospital visit for the D3 and 1 and 3 months thereafter. A SARS-CoV-2 IgG II Quant Kit (Abbott^©^) was used to measure anti-spike (anti-S) and anti-nucleocapsid (anti-N) SARS-CoV-2 IgG concentrations. Previous studies established the utility of this assay: the cut-off values for anti-S and anti-N SARS-CoV-2 IgG were ≥ 50 AU/mL and 1.4, respectively, clearly demonstrating stratification between those recently infected with SARS-CoV-2 and those who had been vaccinated^10^. Age, sex, body mass index, primary kidney disease leading to end-stage renal disease, and past medical history were obtained from the patients’ medical records. Medication information of medications was obtained from prescription records. The results of biochemical measurements, including serum albumin and creatinine, and of complete blood counts were also obtained. The serum ferritin concentration and 25(OH)D, previously shown to be associated with the immune response, were also measured. Serum samples were stored at −80 °C before use. The study was approved by the Ethics Committee of The Jikei University School of Medicine (Approval No. 33-314 [10934]). Written informed consent was obtained from all patients prior to participation in this study. This study was conducted in accordance with the Declaration of Helsinki and its amendments.

### Statistical analysis

The cohort was divided into antibody-positive (≥ 50 AU/mL) and antibody-negative (< 50 AU/mL) groups based on the cut-off value. Patients with a negative anti-S SARS-CoV-2 IgG antibody titer at both 5 months after the D2 and 1 month after the D3 were defined as non-responders. Parametric data are presented as mean and standard deviation (SD), and nonparametric data are presented in interquartile range (IQR). Unpaired t-test, Wilcoxon signed-rank test, Mann–Whitney U test and chi-squared test were used appropriately. Since the dose of mycophenolate mofetil (MMF) varied from patient to patient, MMF was treated as a continuous variable. The risk factors associated with a non-response were evaluated using univariate and multivariate logistic regression models. Anti-S SARS-CoV-2 IgG antibody titers were preprocessed by transforming them to an ordinary logarithm (log10) to adjust for the normality of the distribution. If the distribution of the outcome in the explanatory variables was widely distributed, it was excluded from the analysis. Risks for the two groups were evaluated with odd ratios (ORs) and 95% confidence intervals (CIs). Differences with two-sided *p*-values < 0.05 were considered significant. The data were analyzed using Stata 17.0 (Stata Corp LP, College Station, TX).

## Results

Table 1 lists the characteristics of the 82 kidney transplant recipients (FSGS, focal segmental glomerulosclerosis; TSAT, transferrin saturation; ACE-I, angiotensin-converting enzyme inhibitor; ARB, angiotensin II receptor blocker). The mean age was 53.5 ± 11.6 years, 64.6% were male, and the mean time since kidney transplantation was 10.6 years (IQR 4.0–16.2). The serum creatinine level was 1.2 mg/dL (IQR 1.0–1.8). The BNT162b2 and mRNA-1273 vaccination rates were 85.4% (n = 70) and 14.6% (n = 12) for the D1 and D2, respectively, and 52.4% (n = 43) and 47.6% (n = 39) for the D3.

**Table 1:** Clinical characteristics of the 82 kidney transplant recipients.

Variable	Total (N = 82)
Age, year	53.5 ± 11.6
Sex, male, n (%)	53 (64.6)
Body mass index, kg/m^2^	22.7 ± 3.5
Time post last transplantation, year	10.6 (4.0 to 16.2)
First transplant, n (%)	79 (96.3)
Donor type, living, n (%)	73 (89.0)
Donor age, year	57.5 (49 to 64)
Donor sex, male, n (%)	28 (36.4)
Primary kidney disease	
IgA nephrology	27 (32.9)
FSGS	7 (8.5)
Diabetes mellitus	4 (4.9)
Others	44 (53.7)
ABO incompatible, n (%)	13 (15.9)
HLA class I mismatch	1.7 ± 0.9
HLA class II mismatch	0.9 ± 0.5
Rituximab use, n (%)	24 (29.3)
Methylprednisolone use, n (%)	68 (82.9)
Tacrolimus use, n (%)	71 (86.6)
Mycophenolic acid use, n (%)	74 (90.2)
Mycophenolic acid dose, mg/day	978.7 ± 441.3
Methylprednisolone + Tacrolimus + Mycophenolic acid, n (%)	58 (70.7)
Hemoglobin, g/dL	13.1 ± 2.0
Lymphocyte, /μL	1498.8 ± 668.6
Albumin, g/dL	4.2 ± 0.3
Serum creatinine, mg/dL	1.2 (1.0 to 1.7)
eGFR, mL/min/m^2^	44.9 ± 17.1
TSAT, %	32.6 ± 11.9
Ferritin, ng/mL	119 (57 to 198)
25(OH)D, ng/mL	14.9 (10.6 to 21.3)
Iron drug use, n (%)	13 (15.9)
1α-OH-D3 drug use, n (%)	33 (40.2)
Hypertension, n (%)	56 (68.3)
Diabetes mellitus, n (%)	21 (25.6)
ACE-I or ARB use, n (%)	47 (57.3)
Rejection, n (%)	21 (25.6)
Twice Vaccine type, BNT162b2 vs mRNA-1273, n (%)	70 (85.4) vs 12 (14.6)
Third Vaccine type, BNT162b2 vs mRNA-1273, n (%)	43 (52.4) vs 39 (47.6)

**Figure 2:**
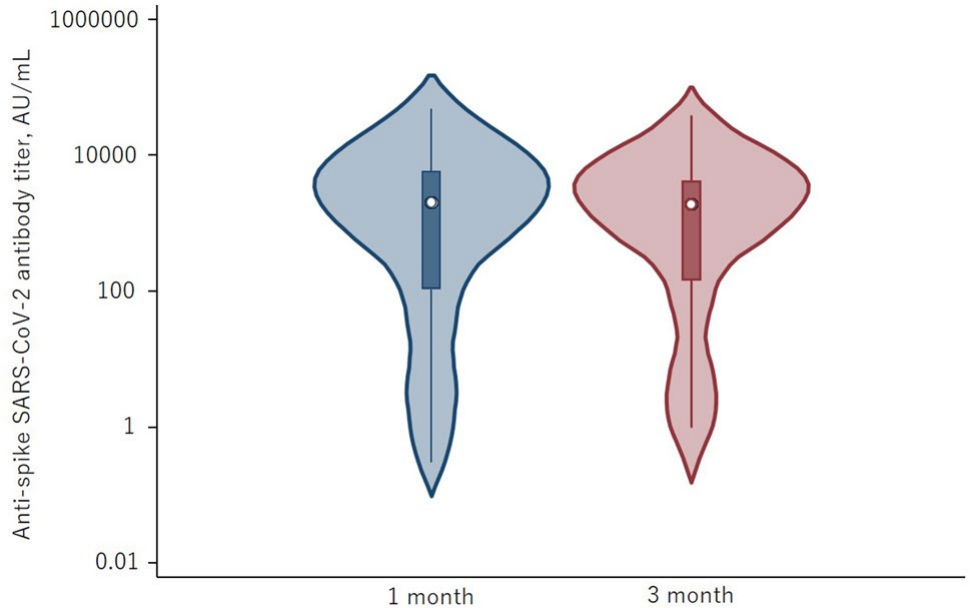
Violin plot of anti-S SARS-CoV-2 IgG antibody titers. Anti-S SARS-CoV-2 IgG antibody titers at 1 month and 3 months. The anti-S antibody titers at 1 month were higher than those at 3 months (*p* < 0.0001).

**Figure 3:**
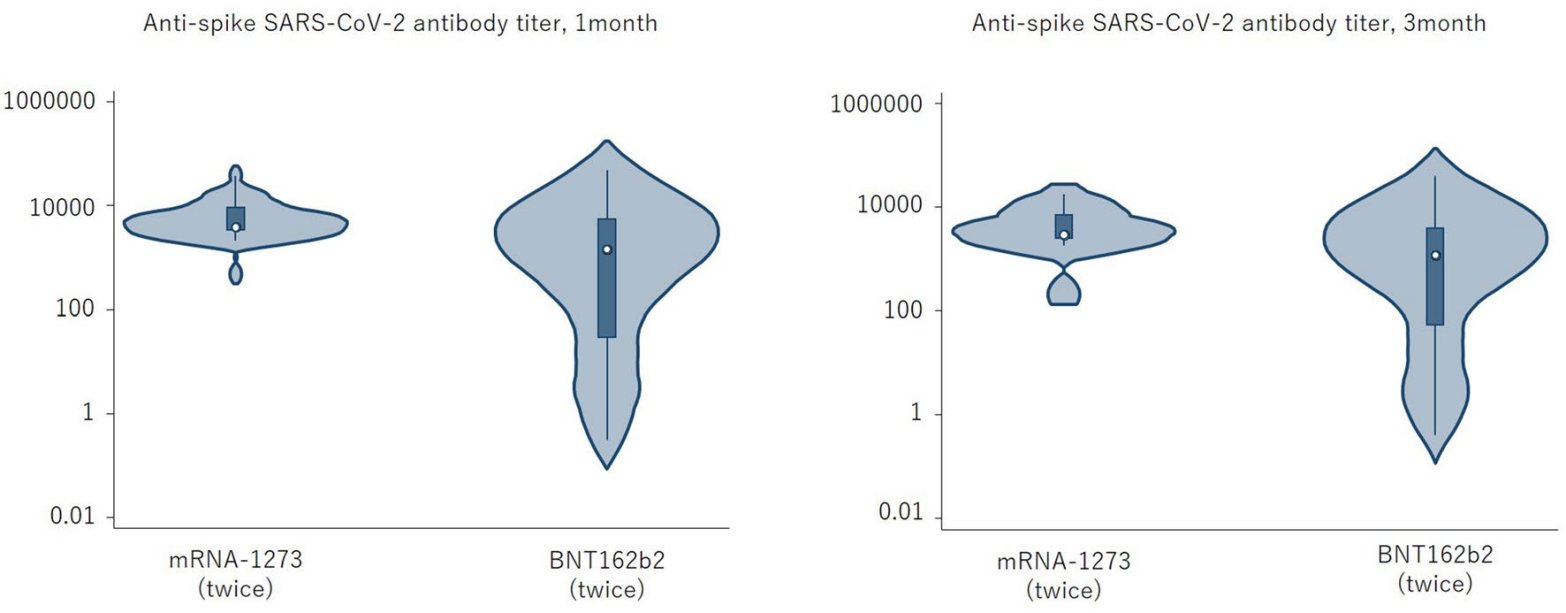
Comparison of anti-S SARS-CoV-2 IgG antibody titers by vaccine type (BNT162b2 or mRNA-1273) at the 1st and 2nd vaccinations. Significantly higher anti-S SARS-CoV-2 IgG antibody titers were measured when the 1st and 2nd vaccinations were mRNA-1273 rather than BNT162b2.

Overall, the anti-S SARS-CoV-2 IgG antibody titers 1 and 3 months after the D3 were 1523.6 AU/mL (IQR 28.4–5612.2) and 1775.4 AU/mL (IQR 52.6–4066.3) (*p* < 0.0001), indicating a decrease over time (Fig. 2). With a cut-off value of ≥ 50.0 AU/mL, the seropositivity rates at 1 and 3 months were 74.7% [negative: 1.35 AU/mL (IQR 0.35–10.6), positive: 3087 AU/mL (IQR 1142.7–8688.2)] and 76% [negative: 1.5 AU/mL (IQR 0.4–4.7), positive: 2544.5 AU/mL (IQR 1069.6–6718.7)]. A comparison of the booster effect of the D3 showed no significant difference in anti-S SARS-CoV-2 IgG antibody titers in patients administered BNT162b2 vs. mRNA-1273 [1 month: 2791.1 AU/mL (IQR 464.2–5320.7) vs. 938.4 AU/mL (IQR 4.9–5612.2), *p* = 0.13; 3 months: 2262.3 AU/mL (IQR 204.2–4066.3) vs. 1050.7 (IQR 4.1–3807), *p* = 0.21]. However, significantly higher anti-S SARS-CoV-2 IgG antibody titers were obtained when the D1 and D2 consisted of the mRNA-1273 vaccine rather than the BNT162b2 vaccine [1 month: 3778.5 AU/mL (IQR 3246.7–9307.5) vs. 1142.7 AU/mL (IQR 19.2–5320.7), *p* = 0.007; 3 months: 2911 AU/mL (IQR 2405.4–7112.4) vs. 1142.7 AU/mL (IQR 11.1–3325.3), *p* = 0.019] (Fig. 3). We measured IgG-S antibody titers at month 1 and month 3 after the third dose of SARS-CoV-2 mRNA and defined the reduction rate as follows: The reduction rate of anti-S SARS-CoV-2 IgG antibody titers = (anti-S SARS-CoV-2 IgG antibody titers at 1 month after D3—anti-S SARS-CoV-2 IgG antibody titers at 3 month after D3)/(anti-S SARS-CoV-2 IgG antibody titers at 1 month after D3) × 100. The reduction rate of anti-S SARS-CoV-2 IgG antibody titers did not significantly differ between BNT162b2 and mRNA-1273 (*p* = 0.41). The anti-N SARS-CoV-2 IgG antibody titers in all patients were < 1.4.

Among the 38 seronegative KTR 5 months after the D2, 25 (65.8%) received BNT162b2 and 13 (34.2%) received mRNA-1273 as D3. With BNT162b2, 11 of 25 (44%) patients became positive, while 14 (56%) remained negative. On the other hand, 7 of 13 patients (53.8%) became positive for mRNA-1273, while 6 (46.2%) remained negative. Thus, 18 (47.4%) became seropositive after the D3 and the non-responder rate was 24.4% (n = 20). Table 2 shows the clinical characteristics of the non-responders (n = 20) and responders (n = 62). Non-responders had a shorter time post last transplantation, higher methylprednisolone use, a higher MMF dose, a lower hemoglobin level and lymphocyte count, a higher serum creatinine level, and higher iron and 1α-OH-D3 use. There was no significant difference between the two groups in terms of history of rejection, with 30% of the non-responder patients and 24.2% of the responder patients. Fortunately, no patient presented with rejection before or after vaccination during this study (Table 2). Logistic regression analyses were performed to identify the factors associated with a non-response. The variables that were significantly different in the univariate analyses were included in the multivariate analysis. The latter showed that a shorter time post last transplantation (OR 0.85, 95% CI 0.75–0.96), a higher MMF dose (OR 1.27, 95% CI 1.06–1.46), a lower hemoglobin level (OR 0.51, 95% CI 0.27–0.94), and a lower lymphocyte count (OR 0.997, 95% CI 0.995–0.999) were associated with a non-response. Mycophenolic acid doses were analyzed at 1/100. (Table 3).

**Table 2:** Clinical characteristics of the non-responder and responder groups.

Variable	IgG S antibody Non-responders (N = 20)	IgG S antibody Responders (N = 62)	*P*-value
Age, year	55.0 ± 9.0	53.1 ± 12.4	0.52
Sex, male, n (%)	12 (60.0)	41 (66.1)	0.62
Body mass index, kg/m^2^	21.5 ± 3.8	23.0 ± 3.3	0.07
Time post last transplantation, year	4.2 (1.7 to 11.5)	11.7 (7.8 to 16.6)	0.006
First transplant, n (%)	18 (90.0)	61 (98.4)	0.08
Donor type, living, n (%)	20 (100.0)	53 (85.5)	0.07
Donor age, year	63.5 (57.5 to 69.5)	55.0 (45.5 to 61.5)	0.0003
Donor sex, male, n (%)	5 (25.0)	23 (40.4)	0.22
Primary kidney disease			0.28
IgA nephrology	10 (50.0)	17 (27.4)	
FSGS	2 (10.0)	5 (8.1)	
Diabetes mellitus	1 (5.0)	3 (4.8)	
Others	7 (35.0)	37 (59.7)	
ABO incompatible, n (%)	2 (10.0)	11 (17.7)	0.68
HLA class I mismatch	1.8 ± 0.6	1.7 ± 1.0	0.93
HLA class II mismatch	0.9 ± 0.5	0.9 ± 0.5	0.77
Rituximab use, n (%)	6 (30.0)	18 (29.0)	0.93
Methylprednisolone use, n (%)	20 (100.0)	48 (77.4)	0.02
Tacrolimus use, n (%)	17 (85.0)	54 (87.1)	0.81
Tacrolimus trough concentration	3.9 (3.2 to 4.9)	4.0 (3.2 to 5.0)	0.70
Mycophenolic acid dose, mg/day	1225.0 ± 404.7	899.2 ± 425.8	0.004
Methylprednisolone + Tacrolimus + Mycophenolic acid, n (%)	16 (80.0)	42 (67.7)	0.30
Hemoglobin, g/dL	11.9 ± 1.3	13.5 ± 2.0	0.001
Lymphocyte, /μL	955.0 ± 424.9	1674.2 ± 639.7	< 0.0001
Albumin, g/dL	4.1 ± 0.4	4.2 ± 0.3	0.40
Serum creatinine, mg/dL	1.7 (1.1 to 2.7)	1.2 (0.9 to 1.5)	0.03
eGFR, mL/min/m^2^	34.6 ± 14.4	48.2 ± 16.6	0.002
TSAT, %	33.3 ± 13.7	32.4 ± 11.3	0.78
Ferritin, ng/mL	123.0 (90.5 to 151.0)	119.0 (52.0 to 205.0)	1.00
25(OH)D, ng/mL	15.7 (10.3 to 21.1)	14.9 (10.7 to 22.2)	0.73
Iron drug use, n (%)	9 (45.0)	4 (6.5)	< 0.001
1α-OH-D3 drug use, n (%)	14 (70.0)	19 (30.7)	0.002
Hypertension, n (%)	13 (65.0)	43 (69.4)	0.72
Diabetes mellitus, n (%)	4 (20.0)	17 (27.4)	0.51
ACE-I or ARB use, n (%)	10 (50.0)	37 (59.7)	0.45
Rejection, n (%)	6 (30.0)	15 (24.2)	0.61
Twice Vaccine type, mRNA-1273 vs BNT162b2, n (%)	0(0) vs 20 (100)	12 (19.4) vs 50 (80.7)	0.03
Third Vaccine type, mRNA-1273 vs BNT162b2, n (%)	6 (30.0) vs 14 (70.0)	33 (53.2) vs 29 (46.8)	0.07

*FSGS* focal segmental glomerulosclerosis, *TSAT* transferrin saturation, *ACE-I* angiotensin-converting enzyme inhibitor, *ARB* angiotensin II receptor blocker. Values are n (%) or presented as means ± standard deviation or medians (interquartile range, 25–75th percentile).

**Table 3:** Factors associated with the lack of a response as determined in univariate and multivariate logistic regression models.

Variable	Univariate Odd ratio (95% CI)	*P* value	Multivariate Odd ratio (95% CI)	*P* value
Age, year	1.01 (0.97 to 1.06)	0.52		
Male gender	0.77 (0.27 to 2.12)	0.62		
Body mass index, kg/m^2^	0.87 (0.74 to 1.02)	0.08		
Time post last transplantation, year	0.90 (0.83 to 0.98)	0.02	0.85 (0.75 to 0.96)	0.01
Treatment with rituximab	1.05 (0.35 to 3.15)	0.93		
Mycophenolic acid dose, mg/day∗	1.25 (1.06 to 1.46)	0.006	1.27 (1.02 to 1.60)	0.04
Tacrolimus	0.84 (0.20 to 3.52)	0.81		
Hemoglobin, g/dL	0.63 (0.46 to 0.86)	0.004	0.51 (0.27 to 0.94)	0.03
Lymphocyte, /μL	0.997 (0.996 to 0.999)	< 0.001	0.997 (0.995 to 0.999)	0.003
eGFR, mL/min/1.73m^2^	0.95 (0.92 to 0.98)	0.003	0.96 (0.90 to 1.02)	0.17
Ferritin, ng/mL	1.00 (0.99 to 1.00)	0.47		
25(OH)D, ng/mL	0.99 (0.92 to 1.05)	0.67		
Hypertension	0.82 (0.28 to 2.38)	0.72		
Diabetes mellitus	0.66 (0.19 to 2.26)	0.51		
Vaccine type, third	2.66 (0.90 to 7.81)	0.08		

∗Mycophenolic acid doses were analyzed at 1/100.

The 11 patients who developed COVID-19 were further examined. In five patients (45.4%) the D1 and D2 consisted of BNT162b2 and in six patients (54.6%) they consisted of mRNA-1273. Overall, in the 11 patients, the anti-S SARS-CoV-2 IgG antibody titer 5 months after the D2 was 11.3 AU/ mL (IQR 3–194.9) [11.3 AU/mL (IQR 3.0–125.2) for BNT162b2 and 61.7 AU/mL (IQR 6.8–194.9) for mRNA-1273]. In total, 2 of the 11 patients were managed at home while the remaining 9 were managed as inpatients. To prevent severe disease, sotrovimab was administered to four patients, molnupiravir to three patients, and nirmatrelvir/ritonavir to one patient. One patient died during the very early period of the COVID-19 pandemic in Japan, when no effective therapy was available. In this case, the second vaccine type was BNT162b2 and died before the D3. The others had mild disease and were discharged safely. In one patient, the TAC level rose to 50.0 ng/mL on the second day after nilmatorvir/ritonavir administration, and tacrolimus had to be discontinued. Furthermore, the TAC level remained elevated thereafter, and the drug was restarted on day 6 after discontinuation.

## Discussion

In the present study, the D3 was given 6 months after the D2, and the anti-S antibody positivity rates at 1 and 3 months were 74.7% and 76.0%, respectively. The median anti-S SARS-CoV-2 IgG antibody titers were 1523.6 AU/mL (IQR 28.4–5612.2) and 1775.4 AU/mL (IQR 52.6–5612.2), respectively (Fig. 2). Of the 38 patients who were negative after the D1 and D2, 18 (47.4%) eventually became seropositive. A simple comparison of the booster effect of the D3 was difficult because vaccination protocols and methods of antibody titer measurement vary from country to country. For example, in studies from France and Canada, the D3 was administered to KTR 1 and 2 months after the D2, with antibody seropositivity rates of 61.2 and 55%^11,12^, respectively. The seropositivity rate of 74.7% in our patients indicates a better booster effect even after a 6-month interval, but the reason for the difference is unclear. However, longer vaccine intervals have been shown to be more immunologically effective in healthcare workers and dialysis patients^13,14^. The former study shows that longer vaccine dosing intervals lead to a predominance of CD4 + T lymphocytes and significant IL-2 production compared to shorter dosing intervals. The authors speculate that IL-2 may be associated with increased antibody production because it is an important aid in the development of B cells into plasma blasts. In two studies from France reporting a poor humoral response after the D1 and D2, a seroconversion rate of 44–49% 4 weeks after the D2 was determined^15,16^. Similarly, in a report from Austria, seroconversion occurred in 47% of KTR as long as 2–4 months after the D2^17^, consistent with our results (seroconversion rate of 47.4%) and demonstrating that the D3 is effective, even 6 months after the D2.

A higher seropositivity in patients vaccinated with the mRNA-1273 vaccine than with the BNT162b2 vaccine 1 month after the D2 was reported from Germany^18^, Belgium^19^, Austria^20^, and Greece^21^. By contrast, in our patients there was no difference in the seropositivity rate obtained with the two vaccine types after the D3. However, after the D1 and D2, the seropositivity rate was significantly higher in patients vaccinated with mRNA-1273 than with BNT162b2. There are four differences between the BNT162b2 and mRNA-1273 vaccines. First, the doses of the mRNA-1273 vaccine are 100–100–50 µg whereas those of the BNT162b2 vaccine are 30–30–30 µg. Second, the interval between the D1 and D2 is longer for mRNA-1273 (4 weeks) than for BNT162b2 (3 weeks). Third, the lipid composition of the nanoparticles used to package the mRNA differs. Fourth, the BNT162b2 vaccine received faster approval than did the mRNA-1273 vaccine^22^. Given the higher dose and longer interval between the D1 and D2, the mRNA-1273 vaccine is more likely than the BNT162b2 vaccine to produce an immunological response.

Even after the D3, 24.4% (n = 20) of our patients were non-responders. In the multivariate logistic model, the factors associated with the lack of a response included a shorter time post last transplant, a higher MMF dose, a lower hemoglobin level, and a lower lymphocyte count (Table 3). With regard to the time post last transplant, one of the risk factors for non-responder in our results was a shorter time post last transplant. Other study demonstrated the same results^23^ but it should be noted that the median observation period in our study was 10.6 years, which is a relatively long time post last transplant, so these results may be limited to recipients with very low immunologic risk. A low MMF dose and low estimated glomerular filtration rate^24^, and a high lymphocyte count^25^ may cause deterioration of the humoral response. Other factors associated with a non-response include older age, steroid pulse therapy, diabetes, a history of cancer, a high serum ferritin level, and a low serum 25(OH)D level^24–26^. Among these risk factors, age, 25(OH)D, and ferritin were examined in this study and were not significant. Similar to our first report^9^, MMF dose was considered to have a strong influence on antibody production in this study. Therefore, it seems important to reevaluate MMF blood levels and optimize MMF dosage. Further analysis of a larger number of patients might reveal the association of these factors.

Methods to induce a response (i.e., an antibody titer to SARS-CoV-2) in non-responders have been examined. In the Netherlands, a randomized controlled trial investigated the use of a higher dose of mRNA vaccine, heterologous vaccination (Ad26. Cov2-S vaccine), and the discontinuation of MMF compared to the usual single additional dose for KTR who remained non-responders despite a second or third mRNA vaccination, but the differences were not statistically significant^27^. However, a group from Baltimore reported that, for the D3, Ad.26.COV2.S was more likely to produce a humoral response in non-responders than was the same BNT162b2/mRNA-1273 vaccination^28^. Due to the current situation, France, Germany, the U.S., and other countries have recommended a fourth and fifth dose for KTR^29–31^, and this may also be a practical approach in Japanese KTR, especially in non-responders.

During the observation period, 11 patients developed COVID-19. In five patients (45.4%) the D3 consisted of BNT162b2 and in six patients (54.6%) it was mRNA-1273. The antibody titer in all 11 patients was very low: 11.3 AU/mL (IQR 3–194.9) 5 months after the D2 [BNT162b2: 11.3 AU/mL (IQR 3.0–125.2); mRNA-1273: 61.7 AU/mL (IQR 6.8–194.9)]. Despite these low antibody titers, the survival rate of the 11 KTR with COVID-19 was excellent because after being diagnosed with COVID-19 in the outpatient clinic most of these patients were admitted to the hospital immediately. Of the 11 KTR, 9 received any COVID-19 therapy such as sotrovimab, molnupiravir, or irmatrelvir/ritonavir. One patient died but the others were discharged safely. Thus, early diagnosis and treatment are crucial for avoiding a high mortality rate in KTR with COVID-19.

The limitations of this study are its single-center design and the sample size of this study is 82 people, which is relatively small, making the results unreliable. In this study, only Japanese people are included. Therefore, it is unclear whether similar results can be obtained for other racial or ethnic groups. Since this study employs an observational study design, it is not possible to confirm a causal relationship. This study followed up only 1 and 3 months after D3 vaccination. Therefore, the long-term antibody response is unknown. One factor affecting non-responder in this study was the shorter time post last transplant, but the relatively long median observation period of 10.6 years in this study may only apply to patients at very low immunologic risk. In addition, cellular immunity was not evaluated. Since the number of KTR who developed COVID-19 was small (11 during the course of the study), the relevance of antibody titers to the development of infection or the prevention of severe disease could not be assessed. Also, the heterogeneity of the antibody titer measurement techniques prevented a quantitative comparison of the results from different studies. In Japan, the policy is to administer a fourth vaccine dose 3 months after the D3. The effects of this additional dose on antibody titers and on the prevention of infection and severe disease remain to be investigated.

In conclusion, a good humoral response was observed in roughly 75% of our KTR after the D3 of an mRNA SARS-CoV-2 vaccine, indicating a sufficient booster effect. However, in 24.4% of patients antibody titers were not detected even after the D3. In these patients, a fourth dose of mRNA SARS-CoV-2 vaccine may be necessary given that KTR are prone to severe disease.

## Data Availability

The datasets used and/or analysed during the current study are available from the corresponding author on reasonable request.
